# Supplementary material for: Quorum Quenchers from Reynoutria japonica in the Battle against Methicillin-Resistant Staphylococcus aureus (MRSA)
Source: Molecules. 2023 Mar 14;28(6):2635. doi: 10.3390/molecules28062635 (PMC10056526; doi:10.3390/molecules28062635)
Supplement: Supplementary file 1 [file molecules-28-02635-s001.zip › molecules-2231875-supplementary.docx]

**Supplementary materials**

# Quorum Quenchers from *Reynoutria japonica* in the Battle of Methicillin Resistant *Staphylococcus aureus* (MRSA)

Maliha Fatima^1^, Arshia Amin^1^, Metab Alharbi^2^, Sundas Ishtiaq^1^, Wasim Sajjad^3*^, Faisal Ahmad^4^, Sajjad Ahmad^5^, Faisal Hanif^6^, Muhammad Faheem^3^, Atif khan Khalil^7^

^1^ Department of Biosciences, Capital University of Science and Technology (e-mail@e-mail.com)

^2^ Department of Pharmacology and Toxicology, College of Pharmacy, King Saud University, P.O.Box 2455, Riyadh 11451, Saudi Arabia.

^3^ Department of Biological Sciences, national University of Medical Sciences 46000

^4^ National Center for Bioinformatics Quaid-i-Azam University Islamabad Pakistan 45320

^5^ Department of Health and Biological Sciences, Abasyn University, Peshawar 25000, Pakistan.

^6^ Department of Microbiology Military Hospital, National University of Medical Sciences 46000

^7^ Department of Pharmacology Lahore College for Women University Punjab 54000

[maliha.fatima.9988@gmail.com](mailto:maliha.fatima.9988@gmail.com), [arshia.butt@cust.edu.pk](mailto:arshia.butt@cust.edu.pk), [mesalharbi@ksu.edu.sa](mailto:mesalharbi@ksu.edu.sa), [sundasishtiaq21@gmail.com](mailto:sundasishtiaq21@gmail.com), [faisalahmad@bs.qau.edu.pk](mailto:faisalahmad@bs.qau.edu.pk), [sajjademaan8@gmail.com](mailto:sajjademaan8@gmail.com), [faisal.hanif@numspak.edu.pk](mailto:faisal.hanif@numspak.edu.pk), [muhammad.faheem@numspak.edu.pk](mailto:muhammad.faheem@numspak.edu.pk), [atif.khalil@lcwu.edu.pk](mailto:atif.khalil@lcwu.edu.pk)

***** Correspondence: [sajjadw@numspak.edu.pk](mailto:sajjadw@numspak.edu.pk) ; Tel.: +92-51-927-0677

**Table. S1** Distribution properties of ligands

| S.N  O | Ligands  Name | VDss  (Huma  n)  (L/kg) | Fraction  unbound (Hum  an)  (Fu) | BBB  Permeabi lity(Hum an)  (Log BB) | CNS Perme  ability  (Log  PS) |
| --- | --- | --- | --- | --- | --- |
| 1 | 2-methoxy-6-acetyl-7-methyljuglone | -0.13 | 0.418 | -0.209 | -2.203 |
| 2 | Emodin | 0.313 | 0.159 | -0.861 | -2.304 |
| 3 | Emodin 8o-b glucoside | 0.488 | 0.195 | -  1.251 | -4.376 |
| 4 | Polydatin | 0.103 | 0.177 | -0.994 | -3.862 |
| 5 | Resveratrol | 0.022 | 0.089 | -0.152 | -2.113 |
| 6 | Physcion | 0.206 | 0.133 | -0.035 | -2.278 |
| 7 | Citreorosein | 0.313 | 0.181 | -0.996 | -3.295 |
| 8 | Quercetin | 0.057 | 0.051 | -1.363 | -3.313 |
| 9 | Hyperoside | 0.899 | 0.165 | -1.539 | -4.721 |
| 10 | Coumarin | -0.128 | 0.344 | -0.013 | -1.992 |

**Table. S2** Metabolic properties of ligands

| S.No | Ligands Name | CYP-2D6 Substrate | CYP-3A4 Substrate | CYP-2D6  Inhibitor | CYP-  2619  Inhibitor | CYP-269  Inhibitor | CYP-2D6  Inhibitor | CYP-3A4  Inhibitor |
| --- | --- | --- | --- | --- | --- | --- | --- | --- |
| 1 | 2-methoxy-6-acetyl-7-methyljuglone | No | No | No | No | No | No | No |
| 2 | Emodin | No | No | Yes | No | No | No | No |
| 3 | Emodin 8-o-b glucoside | No | No | No | No | No |  |  |
|  |  |  |  |  |  |  | No | No |
| 4 | Polydatin | No | No | No | No | No | No | No |
| 5 | Resveratrol | No | No | Yes | No | No | No | No |
| 6 | Physcion | No | No | No | Yes | No | No | No |
| 7 | Citreorosein | No | No | No | No | No | No | No |
| 8 | Quercetin | No | No | Yes | No | No | No | No |
| 9 | Hyperoside | No | No | No | No | No | No | No |
|  |  |  |  |  |  |  | No | No |
| 10 | Coumarin | No | No | Yes | No | No |  |  |

**Table. S3** Excretory properties of ligands

| **S.NO** | **Ligands**  **Name** | **Total Clearance**  **(ml/Kg)** | **Renal OCT2**  **Substrate** |
| --- | --- | --- | --- |
| 1 | 2-methoxy-6-acetyl-7-methyljuglone | 0.022 | No |
| 2 | Emodin | 0.352 | Yes |
| 3 | Emodin 8-o-b glucoside | 0.023 | No |
| 4 | Polydatin | 0.144 | No |
| 5 | Resveratrol | 0.094 | No |
| 6 | Physcion | 0.431 | No |
| 7 | Citreorosein | 0.386 | No |
| 8 | Quercetin | 0.457 | No |
| 9 | Hyperoside | 0.435 | No |
| 10 | Coumarin | 0.96 | No |

**Table. S4** Toxicity properties of ligands

| S.N o | Toxicity Properties | 2-  metho xy-6-  acetyl  -7-  methy ljuglo ne | Emodi n | Emodin  8-o-b glucosi de | Pol yda tin | Resv eratr ol | Phys cion | Citre orose in | Quer  cetin | Hyp eros ide | Coum arin |
| --- | --- | --- | --- | --- | --- | --- | --- | --- | --- | --- | --- |
| 1 | Max  tolerated dose  (Human)  (mg/kg ) | 0.658 | 0.166 | 0.331 | 0.37 4 | 0.561 | 0.293 | 0.205 | 0.742 | 0.50  8 | 0.253 |
| 2 | hERG1  Inhibitor | No | No | No | No | No | No | No | No | No | No |
| 3 | hERG II inhibitor | No | No | No | No | No | No | No | No | Yes | No |
| 4 | Oral rat acute  toxicity  (mol/kg) | 1.845 | 2.281 | 2.54 | 2.39 7 | 2.216 | 2.152 | 2.178 | 2.516 | 2.62  4 | 2.082 |
| 5 | Oral rat chronic | 2.673 | 1.78 | 3.785 | 3.81 7 | 1.761 |  |  |  |  |  |
|  | toxicity  (mg/kg) |  |  |  |  |  | 1.694 | 1.82 | 2.616 | 4.07 | 1.816 |
| **6** | Hepatoxicity (log ug/L) | Yes | No | No | No | No | No | No | No | No | No |
| **7** | Skin Sensitization | No | No | No | No | No | No | No | No | No | No |
| 8 | *T. pyriformis* activity (log ug/L) | 0.509 | 0.62 | 0.285 | 0.28 5 | 0.982 | 0.629 | 0.569 | 0.303 | 0.28  5 | 0.258 |
| 9 | Minnow  toxicity (log mM) | 2.048 | 1.805 | 2.365 | 0.86 4 | 1.367 | 1.191 | 2.232 | 1.999 | 2.70  6 | 1.074 |
